# MRSA surveillance in a rehabilitation centre with liberal isolation policy

**DOI:** 10.1186/1753-6561-5-S6-P180

**Published:** 2011-06-29

**Authors:** I Monsieur, M Wauters

**Affiliations:** 1 Hygiene team, Rehabilitation Centre Inkendaal, Vlezenbeek, Belgium

## Introduction / objectives

In our hospital with 178 beds the core business is multidisciplinar rehabilitation. Close contacts between staff, patients and their environment are inherent in this process. Isolation in single room in case of MRSA is not advisable in the purpose of social reintegration. Isolation is difficult because of long stay and a limited number of single rooms. Before changing the MRSA isolation policy the hygiene team started their infection control program in 2007 with focus on MRSA.

## Methods

In every admission an MRSA screening of the nose is taken. Clinical samples are also taken from exsudative wounds, sputum and urine on admission as well as during hospitalisation. Strategies to prevent patient-to-patient transmission exist from standard precautions with hand hygiene for all residents, waring gloves, gown and eye protection glasses for contact with blood and body fluids. All health care workers are informed and trained by the hygiene team during information sessions.

In case of single MRSA colonization in the nose chemical decolonisation is done.

Microbiological data from screenings and clinical samples are collected. Differentiation between colonization and infection is made.

## Results

The nosocomial MRSA incidence density in clinical samples in 2008, 2009, 2010 was respectively 0.28; 0.15; 0.15. In 2008 only 6 cases out of 15 were nosocomial infection, in 2009 one out of 6; in 2010 6 out of 8.

## Conclusion

The infection control strategies taken for MRSA-spread in our rehabilitation centre don’t lead to a high incidence density of MRSA clinical samples. Continuous focus is given to information about standard and specific precautions and training sessions for all HCW’s. No single room isolation is done for MRSA positive patient. Chemical decolonisation is done in selective patients.

## Disclosure of interest

None declared.

